# Web service QoS prediction using improved software source code metrics

**DOI:** 10.1371/journal.pone.0226867

**Published:** 2020-01-15

**Authors:** Sarathkumar Rangarajan, Huai Liu, Hua Wang

**Affiliations:** 1 Victoria University, Melbourne, Australia; 2 Swinburne University of Technology, Melbourne, Australia; Charles Sturt University, AUSTRALIA

## Abstract

Due to the popularity of Web-based applications, various developers have provided an abundance of Web services with similar functionality. Such similarity makes it challenging for users to discover, select, and recommend appropriate Web services for the service-oriented systems. Quality of Service (QoS) has become a vital criterion for service discovery, selection, and recommendation. Unfortunately, service registries cannot ensure the validity of the available quality values of the Web services provided online. Consequently, predicting the Web services’ QoS values has become a vital way to find the most appropriate services. In this paper, we propose a novel methodology for predicting Web service QoS using source code metrics. The core component is aggregating software metrics using inequality distribution from micro level of individual class to the macro level of the entire Web service. We used correlation between QoS and software metrics to train the learning machine. We validate and evaluate our approach using three sets of software quality metrics. Our results show that the proposed methodology can help improve the efficiency for the prediction of QoS properties using its source code metrics.

## 1 Introduction

Service-Oriented Architecture (SOA) has become a platform for processing large amounts of information and knowledge to provide essential data and services to the users [1, 2]. Web service is the basic unit for a SOA. Web Services(WS) are loosely coupled application programs designed to support business-to-business interoperability using XML based Simple Object Access Protocol(SOAP) [3, 4]. Developers write and publish Web services at service repositories where users can discover, deploy and orchestrate them according to their business needs.

The success of cloud computing depends heavily on the quality of services provided in wireless terminals with limited computing and storage power such as mobile phones [5–7]. WS are of paramount importance among different kinds of cloud services. Indeed the number of WSs is rapidly increasing every day [8]. According to an open source WS repository “programmableweb.com”, they have 22,367 WSslisted. Due to the proliferation of WSs, there are numerous functionally similar services available [9, 10]. Consequently, searching for WS using the functionality keyword is no longer valid. Therefore, non-functional property such as Quality of Service (QoS) have become pivotal criterion in Web service discovery, selection, recommendation and orchestration [11].

Many QoS-driven Web service selection techniques have been proposed and successfully utilized in SOA [12–14]. However, they use QoS values for the Web services made available by the repositories. The QoS data accessible at service repositories have challenges due to some real-world scenarios as stated below:

1. Web service repository turns into a complex hierarchy in various situations. Therefore, end users would take quite a lot of time to go through enormous QoS records [15].
2. Public service repository such as Universal Description Discovery and Integration (UDDI) may hold untrustworthy QoS information due to lack of monitoring. Thus, they might list unavailable services and outdated QoS information for a user’ query [16].
3. Commercial Web services require users to pay a subscription fee to use. Thus, if a user want to test Web service by themselves end up paying considerable amount of money. Obviously, it is not practically possible for a user to monitor and collect QoS data for all the functionally similar WSs.
4. Some public repositories collect feedback from users to acquire QoS data. However, QoS information is influenced by network and geological factors. As Internet is dynamic and vulnerable, it is not possible to get the same QoS values for different users from diverse locations for a particular service [17, 18].

Even though service-level agreement (SLA) contains QoS parameters of a WS, users are still unsure with what is the quality it actually achieves. Certainly, a fundamental pre-request is to predict the QoS values instead of using the data available at repositories. Coscia JLO, Crasso M, Mateos C, Zunino A, and Misra S [19] found a statistically significant and strong relationship among a number of conventional sources code-level metrics and the catalogue of WSDL level service metrics. Observing software quality metrics is a prevalent methodology to evaluate software maintainability. Each Web service comprises of many micro-level software components such as class, method and package. Therefore, source code metrics are calculated at micro-level and aggregated into macro level to represent the entire software efficiently. We hereby list out some of the common practices in software industries for aggregating source code metrics and its disadvantages [20]:

- Simple average: Calculating the mean of metric results for individual elements of a system might not be efficient enough to represent. Because, it does not express the standard deviation and may mitigate the effects of unwanted values in the generalized result. In other words, average function simply smoothed the results but does not reflect the reality.
- Weighted average: Weight could be used to differentiate less important components from critical components. However, defining the weight is very critical and may introduce problem of its own.
- Statistical aggregation methods: Central tendency measures such as mean, median or standard deviation cannot be trusted due to the highly-skewed distribution nature of software.

Impact of aggregation schemes for source code metrics on Web service QoS prediction remains unexplored. We hypothesize that the metrics aggregation scheme plays a vital role for high correlation between many metrics at the file-level. Furthermore, the performance of QoS prediction models may be negatively affected by the potential loss of information due to summation and aggregation.

In this paper, we investigate the impact of source code metrics aggregation on correlation between QoS attributes and source code metrics. Our investigation will be based on three different sets of quality metrics namely object oriented quality metrics proposed by Chidamber SR, and Kemerer CF [21], Complexity metrics proposed by H.M.Sneed [22] and maintainability suite by Baski and Misra [23].

The remainder of the paper is organized as follows: Section 2 presents the motivation and specifications of the research. Section 3 highlights the related work in the research background. Section 4 briefs about the methodologies used to aggregate code metrics and the configuration of the learning machine. Section 5 explains the experimental set up used to validate the proposed approach and discusses the results on Web service QoS prediction. Finally, Section 6 concludes the paper with vision for future work.

## 2 Problem specification

The aim of the research is to investigate the impact of source code metrics aggregation to predict QoS properties. Chidamber and Kemerer explained source code metrics namely Lines of code, functional abstraction measurement, the coupling between object classes, average method complexity, weighted methods per class, McCabe’s cyclomatic complexity [21]. Coscia JLO, Crasso M, Mateos C, Zunino A, and Misra S introduces the possibility to predict the service interface maintainability or QoS by applying traditional software metrics in service implementation. They used source code metrics as a primary pointers to support the software programmers for developing services with more maintainability [19]. Mateos C, Crasso M, Zunino A, and Coscia JLO introduced an interesting correlation between the anti-patterns in the WSDLs and its object-oriented metrics identified from its source code [24]. Kumar L, Kumar M, and Rath SK presented a learning machine to predict the QoS properties such as reliability, response time, throughput, modularity, interoperability, availability, and testability by exploring the correlation between its object oriented software source code metrics [25].

However, less attention had been paid to the aggregation of source code metrics for the Web services so far. Even though a Web service can act as a single standalone system, it comprises of many methods and classes. Thus, aggregation of different micro-level metric values for each pieces of code helps to obtain a single value for global evaluation of the Web service. Arithmetic mean is the predominantly used aggregation function for most of the performance evaluation metric calculation models. However, most of the software quality metric values are much skewed in nature. Consequently, the simple mean function is not reliable against this kind of distributions. A known approach to reduce this problem is to select a known family of distribution such as log-normal, exponential or negative binomial and aggregate the metric value by fitting its observed parameters. This method however is not viable because whenever a new metric is introduced, we need to repeat the fitting process.

As a response to these challenges, we proposed a methodology to calculate source code metrics using micro level software components. We propose an aggregation model for Web service at micro-level by utilizing an inequality measure Theil index. Theil index is widely used in econometrics to study inequality of welfare or income distribution among various groups of people. Distribution of data in econometrics is very much like the data distribution happens in software engineering. As it is having the potential to summarize a large amount of data, it has been proposed recently as an aggregation scheme for software source code metrics.

To achieve the goal of this study, the objectives will be as follows:

- Extracting the source code metrics for micro level attributes from Web service description file named Web Service Description Language (WSDL).
- Calculating source code metrics values using the micro level software components.
- Identifying the correlation between source code metrics (Example: Weighted methods per class, Lack of cohesion in methods) and QoS properties.
- Automate the prediction of QoS properties using the correlation via Machine learning techniques.

We will extract the class files from the WSDL files obtained from QWS-WSDL dataset using WSDL2Java tool. We will calculate source code metrics at class level. Potential source code metrics will be obtained by feature selection and reduction for each quality of service. Finally, learning machine with various kernels will be trained to predict the QoS properties.

## 3 Related work

### 3.1 Web service & quality of service

In practice, it is very difficult for an end user to obtain QoS information. The user needs to spend large amount of resource, time and cost to invoke and measure QoS for all available Web services. Different users will get dissimilar QoS experiences while using the same Web service due to the dynamic nature of the network environment and geographically distributed locations [26]. Therefore, predicting the QoS properties of a Web service became an important step to be followed in service-oriented systems. Using available QoS values in invocation records to calculate the unavailable or missing QoS parameters is called QoS Prediction [27]. Collaborative Filtering (CF) technique is widely adapted in Web service community due to the success in commercial recommender systems. CF predicts unknown QoS values based on historical user data [28].

Predicted QoS values can be used as additional criteria to rank the matching results during the service discovery and selection process. Top ranked service holds an importance among the other services [29]. In service orchestration, considering the QoS of services is as important as combining functionalities of different services.

### 3.2 Software source code metrics

Mateos C, Crasso M, Zunino A, and Coscia JLO [24] discussed the methods available in code-first Web services to remove unnecessary anti-patterns. The authors worked on the hypothesis that the occurrence of anti-pattern can be avoided using object-oriented source code metrics. To find the occurrence of anti-pattern at WSDL level, they have considered eleven source code metrics such as: Average Parameter Count (APC), Response for the Class (RFC), Abstract Type Count (ATC), Coupling between the objects (CBO), Lack of Cohesion among the Methods (LOCM), Void Type Count (VTC), Cohesion among Methods of Class (CAM), Lack of cohesion in methods Henderson-Sellers version(LCOM3), Total Parameter Count (TPC), Weighted Methods per Class (WMC), and Empty Parameters Methods (EPM) [25, 30]. Mateos C, Crasso M, Zunino A, and Coscia JLO used a real-time Web service dataset to identify the correlation between object-oriented metrics and occurrence of anti-pattern by using well-known statistical methods. They also measured the impact of simple metric-driven code refactoring on the occurrence of anti-pattern to some of the generated WSDLs from the dataset. As a summary, Mateos C, Crasso M, Zunino A, and Coscia JLO observed that the complexity and maintainability of Web services can be predicted using object-oriented metrics and refactoring.

### 3.3 Correlation between source code metrics and QoS

A high correlation between traditional Object-oriented source code-level metrics and WSDL-level service metrics have been found by Charrad et al [29]. They used the most comprehensive and thoroughly evaluated set of metrics to calculate the maintainability of Web service using WSDL interfaces. The findings of this paper suggest that the software developers can avoid developing non-maintainable service by applying simple early code refactoring. As Java is widely used as a programming language to develop back-end services, the authors focused on java based Web services but their finding does not depend on the programming language.

Romano et al. tried to identify the list of source code metrics which can be used to predict Java interfaces that are vulnerable to change [31]. The source code metrics such as Lack of Cohesion among Methods (LCOM), Coupling between Object (CBO), Depth of inheritance tree (DIT), Number of Children (NOC), Weighted Method per Class (WMC), Response for Class (RFC), and Interface Usage Cohesion (IUC) were used along with fine-grained source code changes in interfaces of ten open-source Java-based systems [25, 30]. The correlation between the metrics of the source code and the fine-grained changes in the source code was tested empirically. Romano et al. concluded that the external interface cohesion source metrics have greatest association with the number of changes in source code.

### 3.4 Software metrics aggregation

Software metrics calculated at micro-level artefacts and aggregated to the macro-level artefact for the analysis. The popular aggregation technique used for source code metrics is mean [32], [33] even though there are increasing research works to demonstrate the inappropriateness of this technique [34], [35] due to the skewness of source code metrics distribution [36]. The sum is another popular aggregation technique. Chidamber et al. used the sum to aggregate complexity of individual methods to the class level in their metrics suite [21]. Alexander et al. [37] used the Theil index, a widely used inequality measure in econometrics to identify the wealth distribution to aggregate the software metrics values as they both share the same kind of data distribution. Theil index is not specific to a particular metric and can be used to aggregate a wide range of metrics.

## 4 Proposed work

### 4.1 Research framework

**Fig 1:**
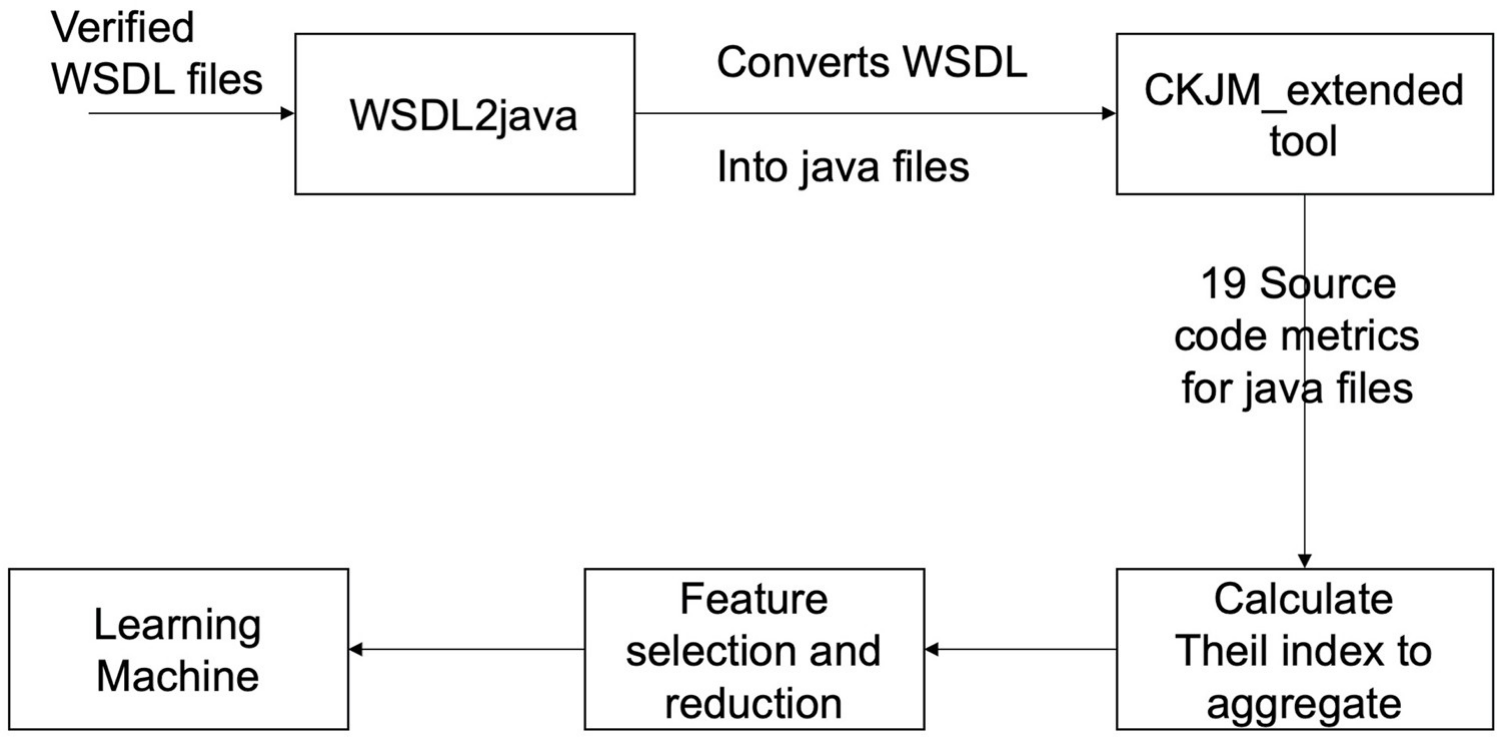
The proposed framework.

Fig 1 demonstrates our proposed structure and methodologies for this research. The system is made up of many steps and the first step is to extract java class files from WSDL files using WSDL2java software. The next step is to measure the various metrics of the source code. By following the steps described in Fig 1, we used CKJM_extended tool to measure the object-oriented source code metrics. For each Java class file, we calculate 19 metrics of source code. Then we use the *Theil* index as an aggregation technique to sum up a single value for all the metrics of the source code. We used Principal Component Analysis to remove irrelevant features to achieve dimensionality reduction. As a final step, we apply linear regression to predict the quality metrics for the Web services. By using the different combination of available source code metric sets, we got seven sets of metrics to evaluate the performance of regression learner. Performance of prediction model using different sets of metrics evaluated by calculating estimator evaluation metrics.

### 4.2 Source code metrics aggregation

Data of wealth distribution inequality from economics and source code metrics of software are sharing similar structure. The Gini coefficient, a widely applied economics inequality measures attracted attention in the field of software metrics. It can be easily explained using Lorenz curve. However, the Gini coefficient has a major drawback as it cannot be decomposed [37]. Serebrenik et al. proposed another inequality measure named Theil index instead of Gini index as it is decomposable so it can be used not only to calculate the inequality but also to explain it. Moreover, it is not specific to any specific metrics so it can be used to aggregate wide range of metrics. [38]. Therefore, we preferred Theil index to calculate the aggregation for source code metrics.

### 4.3 Feature redcution & selection

We used Principal Component Analysis (PCA) as data preprocessing method for feature extraction and selection. For each PC (Principal Components), we calculate eigenvalue, variance percent, cumulative percentage and source code metrics interpreted. PCs with eigen value more than 1 are considered as a potential source code metrics coherts. Table 1 shows the PCA results of object oriented metrics. Out of 19 source code metrics, 12 metrics were identified as having potential to predict QoS properties. Tables 2 and 3 shows the PCA results for Baski & Misra metrics and Sneed’s metrics.

**Table 1:** PCA results for object oriented metrics.

Principal component	Eigenvalue	% Variance	% cumulative	Metric Interpreted
PC 1	5.744292522	38.29528348	38.29528348	WMC, CBO, RFC, Ca, LCOM, Ce, NPM, LOC, MOA, CAM, AMC
PC 2	3.553881589	23.69254393	61.98782741	DIT, CBO, LCOM, Ca, Ce, LOC, DAM, MOA, MFA, CAM, AMC
PC 3	1.92886401	12.8590934	74.8469208	WMC, CBO, RFC, Ca, LCOM, Ce, NPM, AMC
PC 4	1.104911736	7.36607824	82.21299904	WMC, RFC, LCOM, Ca, Ce, NPM, DAM, MOA, MFA, CAM, AMC

**Table 2:** PCA results for Baski & Misra metrics.

Principal component	Eigenvalue	% Variance	% cumulative	Metric Interpreted
PC 1	2.908	48.465	48.465	OPS, DW,MRS,DMC
PC 2	1.489	24.809	73.275	DMC,DMR,ME

**Table 3:** PCA results for Sneed’s metrics.

Principal component	Eigenvalue	% Variance	% cumulative	Metric Interpreted
PC 1	2.359	29.490	29.490	Data flow complexity, Data access complexity, Interface complexity, Control flow complexity, Decisional complexity, Branching complexity, Language complexity
PC 2	1.946	24.327	53.817	Data complexity, Data flow complexity, Decisional complexity, Branching complexity, Language complexity
PC 3	1.320	16.501	70.318	Data flow complexity, interface complexity, Language complexity

### 4.4 Learning machine

The aim of this research is to examine the impact of aggregation methods of source code metrics (e.g. Lack of cohesion in methods, coupling between object classes) on predicting QoS characteristics (e.g. reaction time, accessibility, throughput, testability, interoperability, etc.). Therefore, We preferred to use a simple regression model called multiple linear regression model to employ the prediction. By fitting a linear equation to measured data, multiple linear regression aims to model the relationship between two or more independent variables and a response variable. We developed the training set according to the correlation standards defined in [25]. Then the number of latent variables need to be defined for each QoS property. The next step is to build a model to generate the value of the QoS property by using the training set knowledge base. The data set was submitted to a 10-fold cross-validated paired t-test analysis. In the 10-fold cross-validated paired t-test procedure we segment the dataset into 10 parts of equally sized, each of which is then used for analysis, while the remaining 10-1 parts (joined together) are used to train the regressor (i.e., generic k-fold cross-validation). For demonstrating the reliability of the regression learner, we used two different performance metrics (MAE, RMSE).

## 5 Experiments & analysis of results

### 5.1 Research questions

Our experimental study designed to answer the following two research questions:

#### 5.1.1 RQ1: How does the proposed methodology improve the predictability of source code metrics?

A software system is not a single standalone system to provide the solution. Normally, it comprises many subordinate pieces of code such as class, method or function. Therefore, the software metrics must calculated in the micro level and should aggregated into macro level for representing the source code metric of a software system. Since software code metrics are highly skewed values, it is inappropriate to use simple statistical aggregation (mean, median, etc.,) methods. The proposed inequality distribution models are very successful in economics data, which is as skewed as software source code data.

#### 5.1.2 RQ2: How are source code metrics used to predict quality of service properties of Web service using source code metrics?

During the software development life cycle, developers extract source code metrics to evaluate the maintainability to reduce the future issues with the system. Thus, source code metrics and quality of service properties are correlated. We will use the correlation between source code metrics and quality of service to predict the QoS of Web services. We use linear regression based learning machines to create a prediction model.

### 5.2 Variables and objects

#### 5.2.1 Independent variable

A technique under investigation is defined as independent variable. So, Theil index is selected as the independent variable for this research. Dutch statistician Henri Theil presented an inequality measure named Theil index [39]. Given a (continuous) univariate distribution function *F* with the support X⊆R and the mean *μ*(*F*) the first Theil index is defined as:

$$\begin{array}{c} I_{Theil}\left(F\right)=\int \frac{x}{\mu (F)}log(\frac{x}{\mu (F)})dF\left(x\right) \end{array}$$ (1)

The Theil index was introduced for the field of unequal income distribution aggregation. Let *x*_0_ ∈ *X* is a particular value of income, *F* is a distribution of income in the population, and *F*(*x*_0_) is the proportion of the population with the income *x* less than or equal to *x*_0_. We calculate source code metrics at micro level and aggregate to represent the macro level software using Theil index. Metrics that have negative values can not be aggregated using Theil index because of the logarithmic calculation in its formula. Since *logx* for *x* ≤ 0 can produce an undefined value, Theil index may also be an undefined value if they contains non-positive values [37].

#### 5.2.2 Dependent variable

Mean Absolute Error (MAE) and Root Mean Squared Error (RMSE) are the two well-known statistical precision measurements utilized to assess the prediction results. MAE is the average absolute deviation of predictions to the ground truth data. For all test services and test QoS properties, MAE is calculated as:

$$\begin{array}{c} MAE=\frac{\left(\sum ij\|Q_{ij}-{\hat{Q}}_{ij}\|\right)}{N} \end{array}$$ (2)

In the Eq 2, *Q*_*ij*_ denotes the observed QoS value of Web service *j* obtained from data set entry *i*; ${\hat{Q}}_{ij}$ is the predicted QoS value; and *N* is the number of predicted values. The smaller value of MAE indicates better prediction result. RMSE can be expressed as:

$$\begin{array}{c} RMSE=\sqrt{\frac{\sum ij\|Q_{ij}-{\hat{Q}}_{ij}{\|}^{2}}{N}} \end{array}$$ (3)

RMSE can be measured using Eq 3 to find out the differences between the actual and predicted values. Once the model yields more than 90% accuracy level, the learning machine can able to predict the five chosen QoS properties for a WSDL of a service.

#### 5.2.3 Objects

We used the WS dream dataset, an open source dataset produced by a group of researchers from The Chinese University of Hong Kong. WS dream dataset contains two versions of datasets and we used version 1 for the experiment. The dataset contains URLs of 5825 Web services and its response time and throughput readings from 339 geographically distributed users. The dataset also has more details about both the Web services and users such as IP address, country, continent, longitude, latitude, region, and city.

Our main goal of this research is to identify the implications of aggregation of source code metrics on QoS prediction. Therefore, we need to calculate the source code metrics for the target Web services using its Web Service Description Language (WSDL) is necessary. wsimport is a Java function to process WSDL file and extract class files for the corresponding Web services.

**Fig 2:**
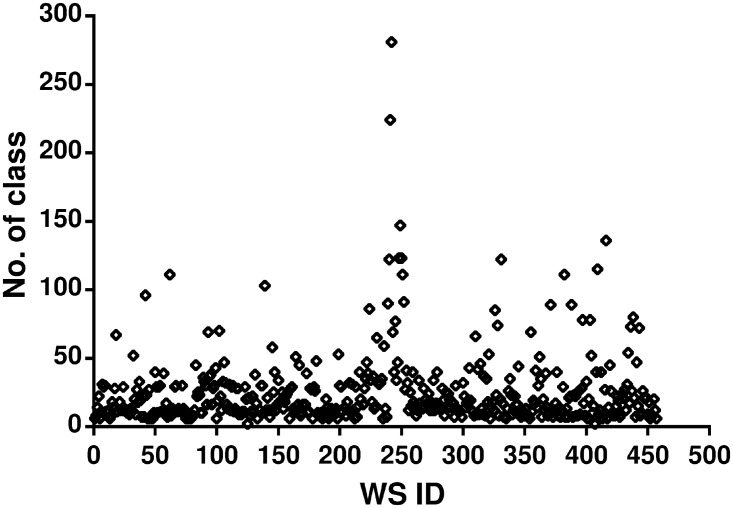
Number of Java files extracted from available WSDL.

Since the dataset only contains URL for the WSDL of Web services, we developed a Java application to crawl the WSDL files using URLs from the dataset. Only 457 out of 5825 Web services have active WSDL available on the internet. As we mentioned earlier, wsimport was used to extract the Java class files. The number of class files per Web services ranges from one to 281. Fig 2 shows the number of class files per Web service. The x-axis represents the Web service ID and y-axis represents the number of Java files extracted from the Web service.

### 5.3 Empirical environment

#### 5.3.1 Source code metrics

We used three sets of metrics sucha as object oriented source code metrics proposed by Chidamber et al. [21], Baski and Misra Metrics [23] and Sneed’s metrics [22].

**Chidamber and Kemerer Metrics**: Ckjm_extended tool introduced by Chidamber et al. [21] can be utilized to calculate 19 size and structure software source code metrics from the generated Java class files. The metrics are Weighted methods per class, Depth of Inheritance Tree, Number of Children, Coupling between object classes, Response for a Class, Lack of cohesion in methods, Afferent coupling, Efferent coupling, Number of Public Methods for a class, Lack of cohesion in methods Henderson-Sellers version, Lines of Code, Data Access Metric, Measure of Aggregation, Measure of Functional Abstraction, Cohesion Among Methods of Class, Inheritance Coupling, Coupling Between Methods, Average Method Complexity, McCabe’s Cyclomatic Complexity.

We developed a Java based system to integrate with ckjm tool and calculated 19 metrics for all the 457 Web services. For the next step, we used R language to calculate the statistical calculation. R library called *ineq* have been used to calculate the Theil index *I*_*Theil*_ for the Web services.

**Sneed’s tool**: Sneed et al. [22] developed a tool named softAudit for measuring Web Service interfaces. The suite consists of nine different source code metrics to measure complexity of service interfaces: Data Complexity (Chapin Metric), Data Flow Complexity (Elshof Metric), Data Access Complexity (Card Metric), Interface Complexity (Henry Metric), Control Flow Complexity (Mccabe Metric), Decisional Complexity (Mcclure Metric), Branching Complexity (Sneed Metric), Language Complexity (Halstead Metric), Weighted Average Program Complexity. We used softaudit tool provided by Sneed et al. to calculate the 09 complexity metrics for all the Web service by processing micro-level class files. We also calculated 09 quality metrics using sneed’s tool namely Degree of Modularity, Degree Of Portability, Degree Of Reusability, Degree Of Testability, Degree Of Convertibility, Degree Of Flexibility, Degree Of Conformity, Degree Of Maintainability, Weighted Average Program Quality.

**Baski and Misra Metrics**: Baski and Misra [23] proposed a tool to compute six different complexity metrics of WSDL file These metrics are based on analyzing the WSDL and XSD schema elements. The metrics are Data Weight of a WSDL (DW), Distinct Message Ratio (DMR), Distinct Message Count (DMC), Message Entropy (ME), Message Repetition Scale (MRS) and Operations Per Service (OPS). Baski & Misra metrics have been calculated by processing the WSDL file for each web service rather than processing micro-level class files. We used Baski & Misra’s tool to calculate the six metrics for the 457 Web services WSDL files.

We used above mentioned three sets of quality metrics as an independent variables and quality metric values calculated using Sneed’s tool as a dependent variable. Normalization is very important to process the data for regression. So, we applied unsupervised normalization with the range of 0.0 to 1.0 using Weka tool. Then we grouped the metric sets with different combination to populate more sets of metrics to compare our results. Consequently, we have Chidamber and Kemerer Metrics (CKM), Baski and Misra Metrics (BSM), Sneed’s metrics (SM), CKM-BSM metrics, CKM-SM metrics, BSM-SM metrics and All metrics (AM). After grouping, we got seven different sets of metrics available. We applied linear regression to predict modularity, quality of service value calculated. The results show that Sneed’s metrics individually outperform the other sets of metrics. Object-oriented metrics also have the potential to predict the QoS value but not as efficient as Sneed’s metrics. Baski & Misra metrics have the lowest efficiency among the available group of metrics. In summary, metric values calculated at the micro-level have better QoS prediction efficiency than those at macro level.

### 5.4 Result analysis

**Fig 3:**
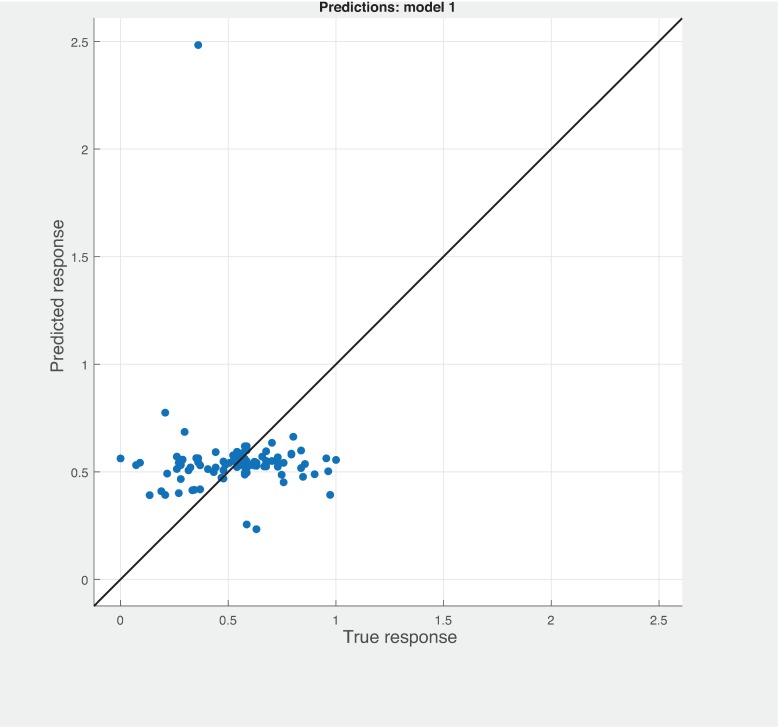
BM metrics vs modularity prediction.

**Fig 4:**
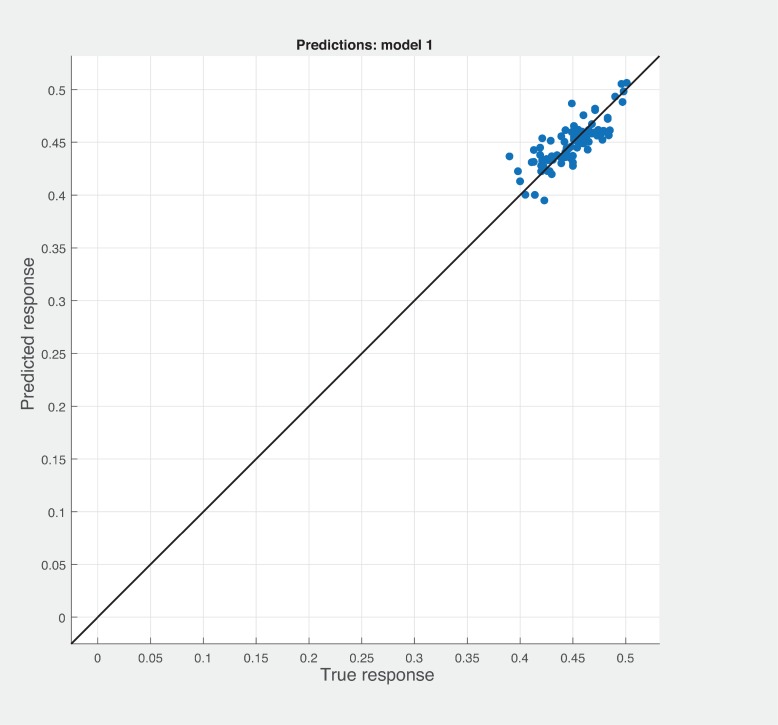
CKJM metrics vs modularity prediction.

**Fig 5:**
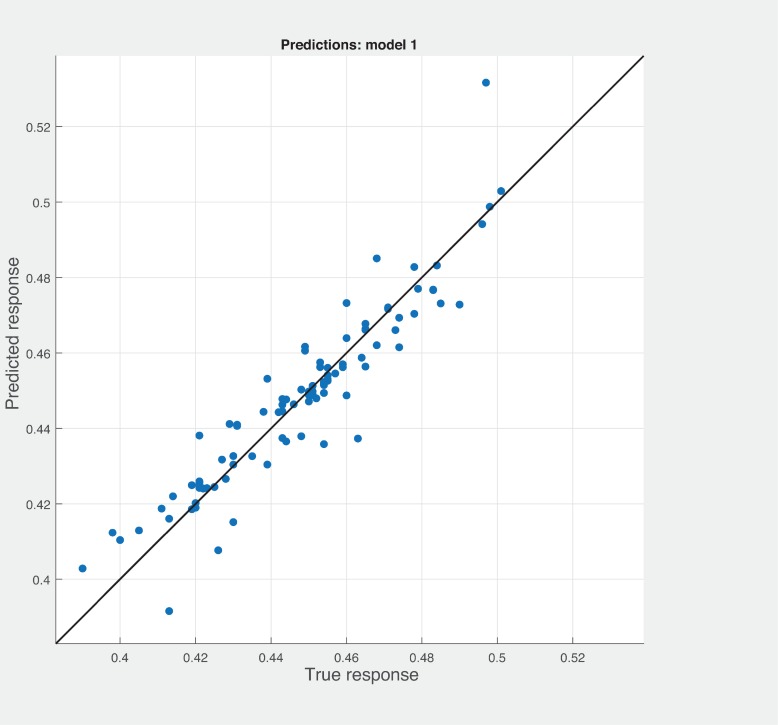
Prediction model for modularity using SM metrics.

Three Quality of service metrics namely Modularity, Testability, Maintainability, Reusability for the available Web service had been calculated using Sneed’ tool. All three sets of metrics and its combinations had been used to predict the metrics value using robust linear regression. Fig 3 shows the graph between actual modularity values and predicted modularity values. The RMSE and MAE values respectively, 0.284, 0.172 which not a better values for a linear prediction model. Fig 4 contains the comparative graph of predicted modularity values using CKJM metrics and actual modularity values. RMSE and MAE values are 0.017, 0.011. The prediction results are better than Baski & Misra metrics. The graph depicted in Fig 5 illustrates the comparative study between actual vs predicted modularity values using sneed’s metrics. The results shows the sneed metrics have higher potential to predict the quality metrics for the Web service compare to other two metrics. The RMSE and MAE values are 0.0085, 0.0057 that implies the prediction results are very good.

**Fig 6:**
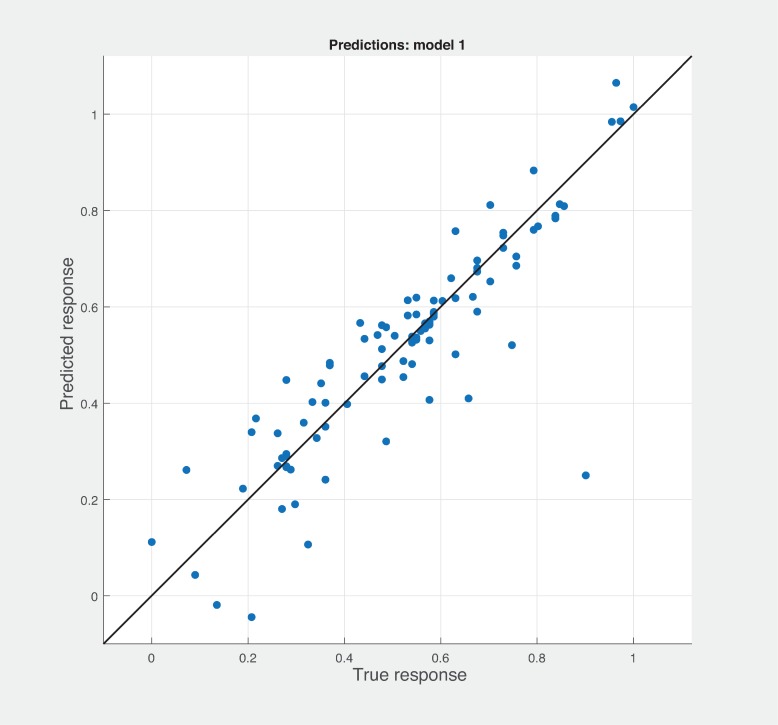
Modularity prediction results for SM-CKM metrics.

**Fig 7:**
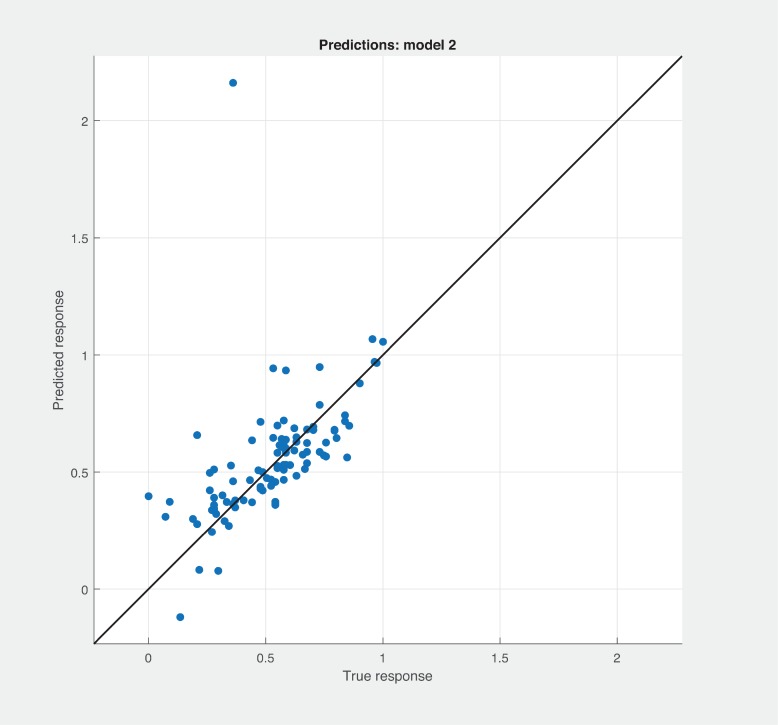
Modularity prediction results with BSM-CKM metrics.

**Fig 8:**
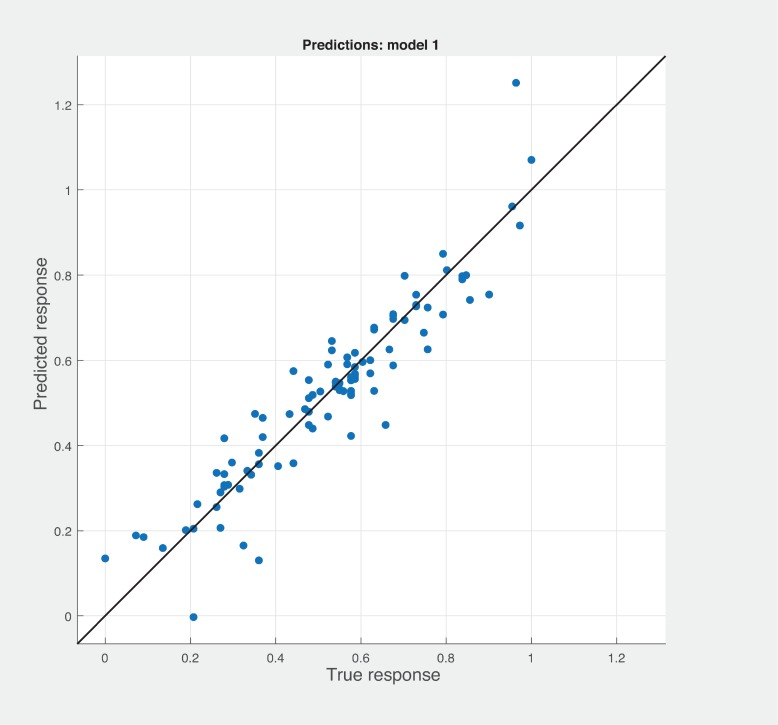
Modularity prediction results with BSM-SM metrics.

**Fig 9:**
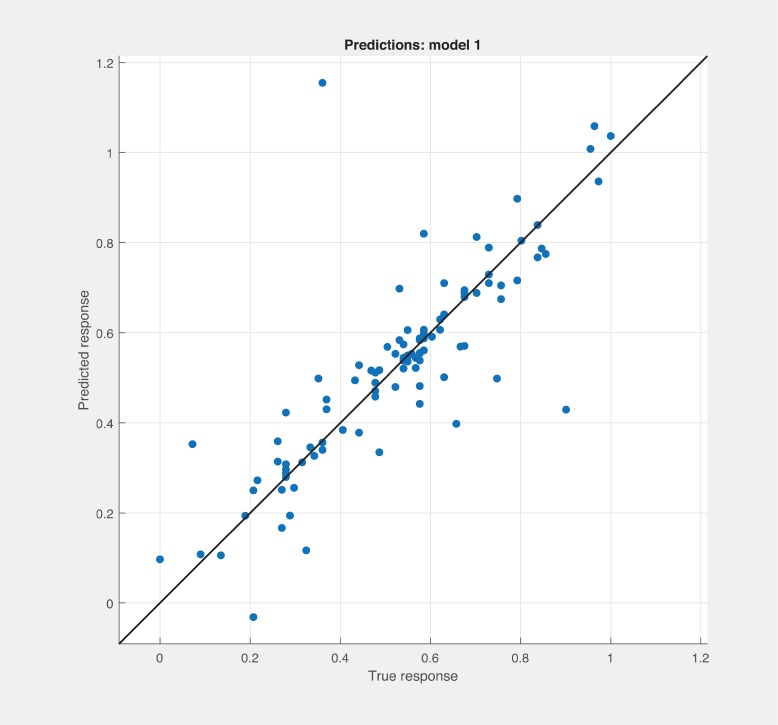
Modularity predicted vs actual for All metrics.

**Fig 10:**
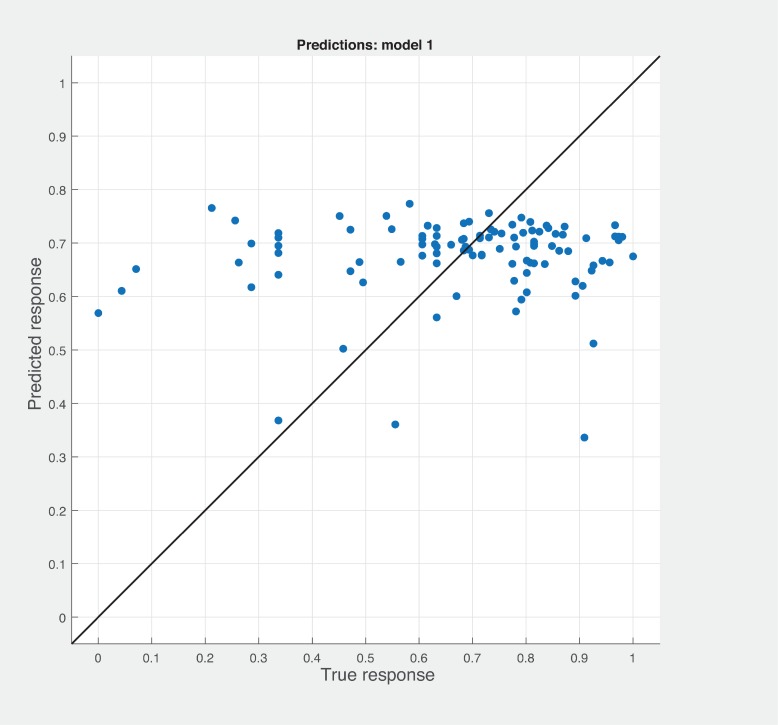
Testability prediction results with BSM metrics.

**Fig 11:**
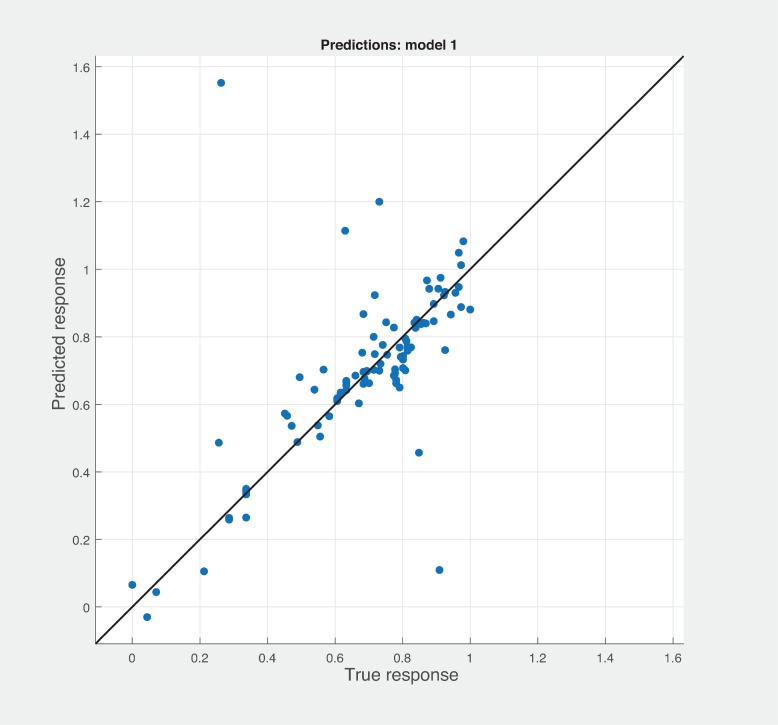
Testability predicted vs actual for all metrics.

**Fig 12:**
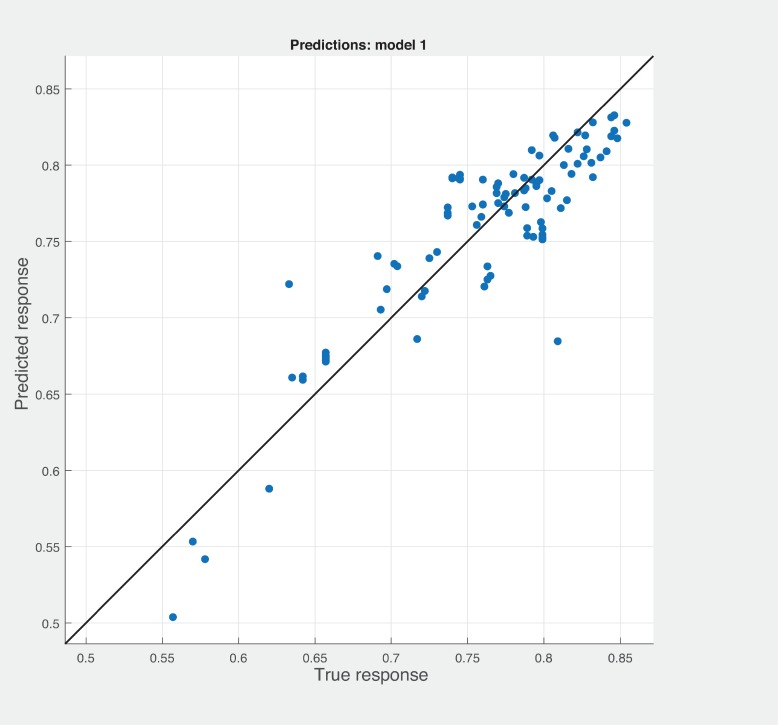
Testability prediction results with Sneed’s metrics.

Figs 6, 7 and 8 shows the prediction results with different combination among the three sets of metrics. Neither combination produced better results than the sneed’ set of metrics. As shown in Fig 7, BSM-CKM metrics have least potential to produce better prediction of modularity. The prediction and actual modularity values using all metrics comparative graph shown in Fig 9. It also could not produce better results than SM metrics. Table 4 shows the summary of RMSE and AME values of different sets of metrics used for the robust linear prediction. Sneed has set of metrics as a stand-alone predictors to produce better results for modularity quality prediction. As stated in Figs 10, 11 and 12, all metrics combined produces slightly better result than BaSki and Misra (BSM) metrics for predicting testability. However, Sneed’s metrics yields way better results than the all metrics and BSM metrics.

**Table 4:** RMSE & MAE comparison for different sets of metrics for modularity.

S.No	Metric set name	RMSE	MAE
1	BSM	0.284	0.172
2	CKM	0.017	0.011
3	SM	0.0085	0.0057
4	BSM-CKM	0.1497	0.022
5	BSM-SM	0.0872	0.0581
6	SM-CKM	0.12114	0.085
7	AM	0.1556	0.0743

**Fig 13:**
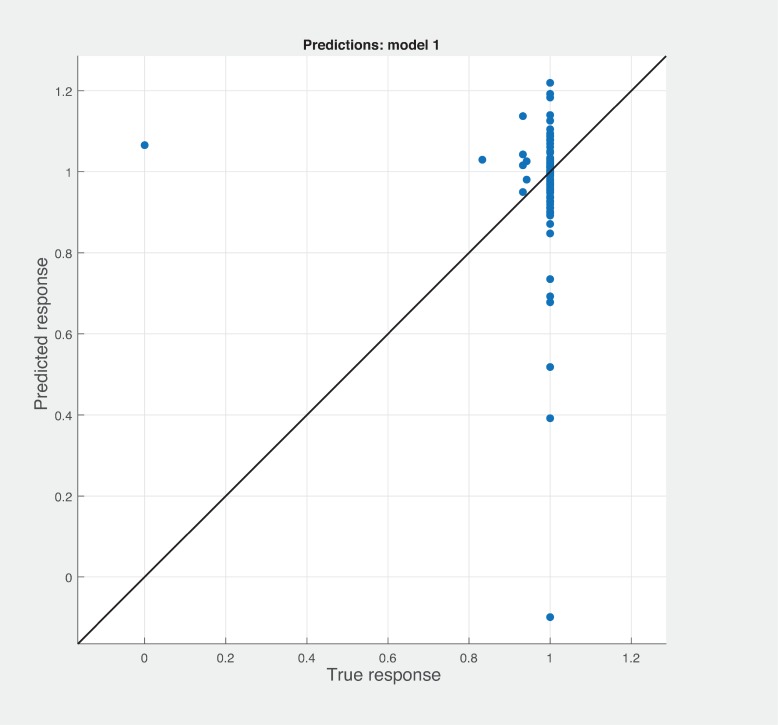
Reusability predicted vs actual for all metrics.

**Fig 14:**
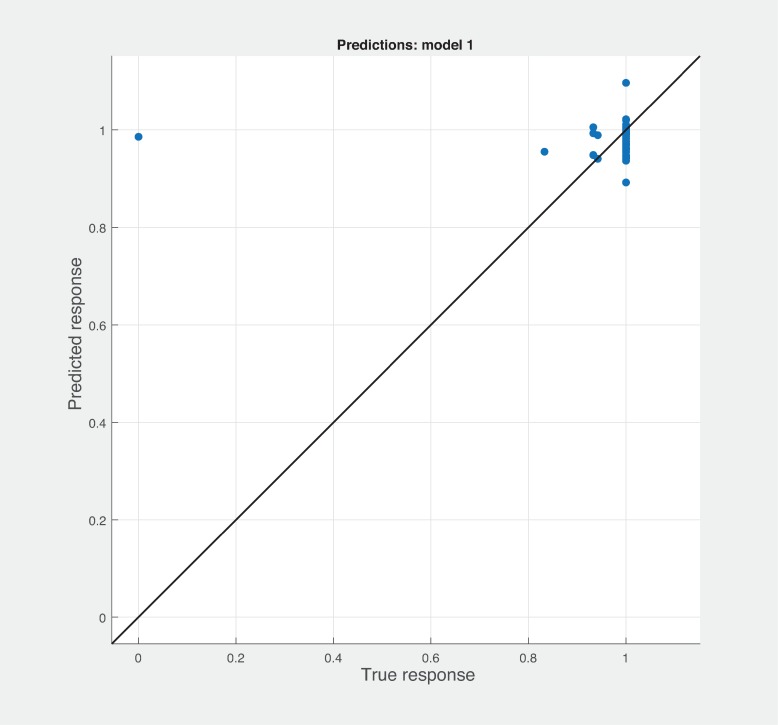
Reusability prediction results with BSM metrics.

**Fig 15:**
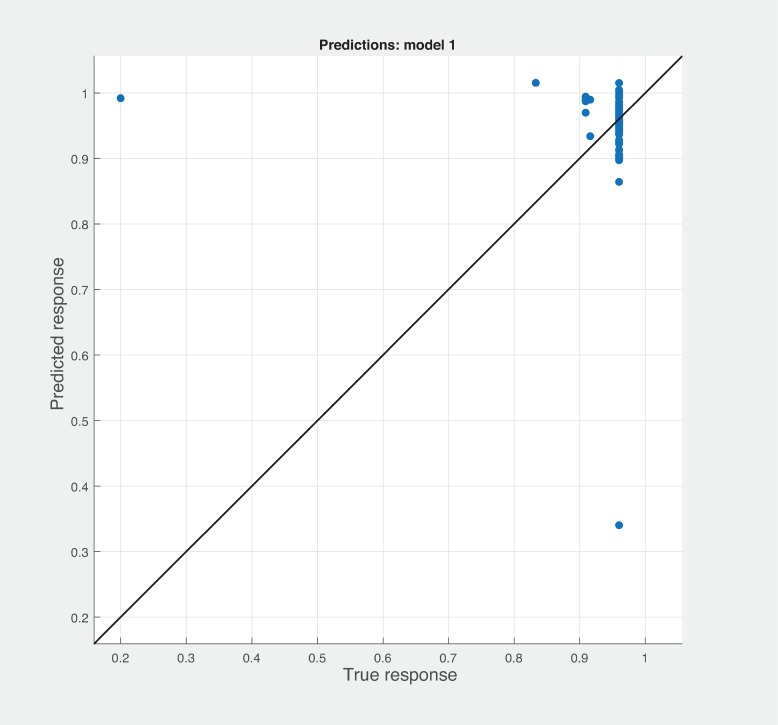
Reusability predicted vs actual for Sneed’s metrics.

**Fig 16:**
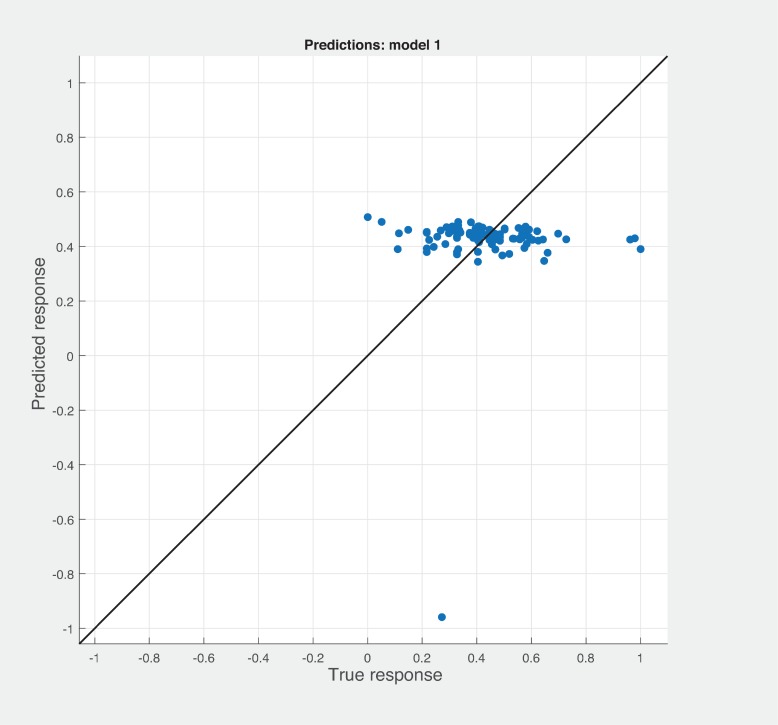
Maintanability prediction results with BSM metrics.

**Fig 17:**
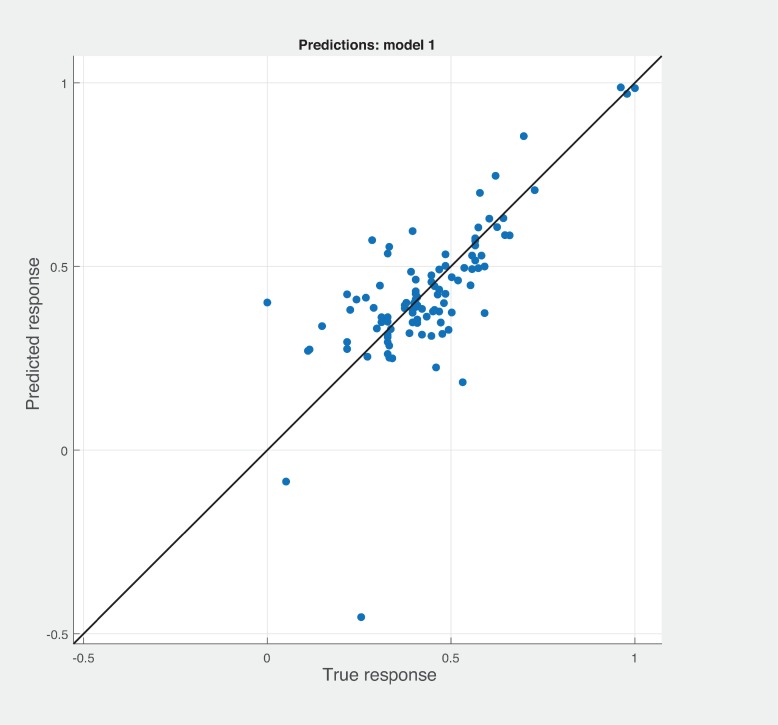
Maintainability predicted vs actual for all metrics.

**Fig 18:**
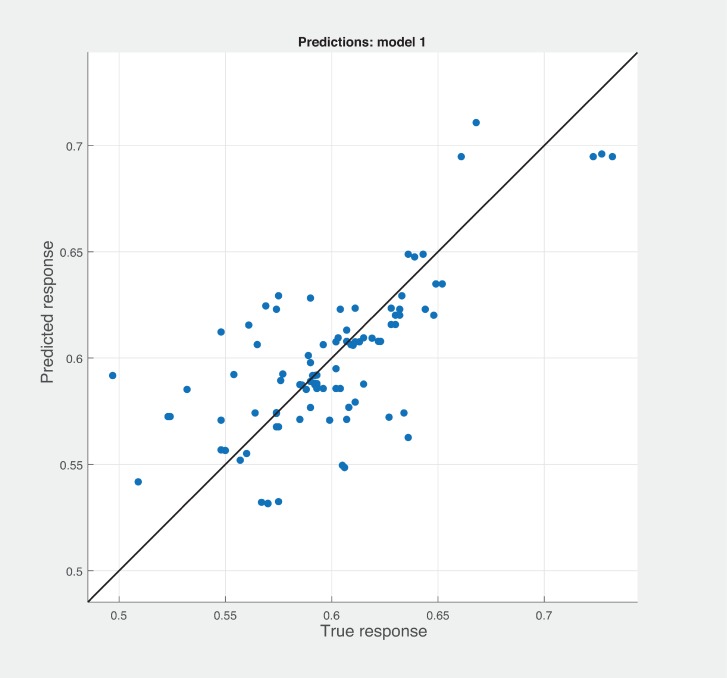
Maintanability prediction results with Sneed’s metrics.

Figs 13, 14 and 15 shows that using all metrics combined and BSM metrics could not achieve better results for Reusability prediction. Sneed’ metrics produces slightly better results while compare with other sets of metrics. By comparing Figs 16 and 17, all metrics together produces better prediction results than BSM metrics. As stated in Fig 18 Sneed’ metrics shows better potential than over all metrics combined and BSM metrics for predicting Maintainability.

The comparison table shows that the CKM metrics and SM metrics individually produce better results. When CKM and SM combined the efficiency of the prediction quality is reduced indicated by the increasing MAE and RMSE values. BSM metrics have lower potential to predict the quality values individually. The results of BSM combined with SM is better than CKM. As a conclusion, we demonstrated that the CKM and SM metrics that are calculated from micro level have higher potential to predict the quality of a Web service.

## 6 Conclusion

QoS values have become an essential criterion to choose a suitable service from abundance of functionally similar Web services. On the other hand, service providers do not provide adequate QoS data, while unequal computing and network environment make the user provided QoS data invalid. Therefore, predicting the QoS values of Web service is an important step in service-oriented systems. One of the independent methods to predict the quality parameters of the Web service is to utilize the software code metrics. Web service is not just a single system, but it contains many classes and methods. Thus, source code metrics should be calculated from the micro level such as classes and methods to a macro level system. However, most of the current systems either calculates the code metrics at a macro level or use basic arithmetic average and mean to lift the metrics from class level to system level. Such methods may cover up the inefficient values and provide the values of a rounded-up metric for the predictor.

In this paper, we investigated the impact of calculating source code metrics from a micro level on predicting the quality metrics. We used three sets of metrics namely CKM, BSM and SM to validate our system. For CKM metrics, we calculated source code metrics at the class level and used the Theil index, a method to aggregate source code metrics without compromising the distributed nature of the software source code. For SM metrics, we used Sneed’s tool to calculate complexity metrics from the Web service class files. BSM metrics have been calculated using its macro-level WSDL file instead of class files. We applied linear regression to predict modularity, quality of service value calculated. The results show that SM metrics individually outperform the other sets of metrics. CKM metrics also have the potential to predict the QoS value but not as efficient as SM metrics. BSM metrics have the lowest efficiency among the available group of metrics. In summary, metric values calculated at the micro level have better QoS prediction efficiency than those at macro level.
